# Precipitate Evolution in 22Cr25NiWCuCo(Nb) Austenitic Heat-Resistant Stainless Steel during Heat Treatment at 1200 °C

**DOI:** 10.3390/ma14051104

**Published:** 2021-02-26

**Authors:** Sheng-Min Yang, Jing-Lin Wu, Yeong-Tsuen Pan, Dong-Yih Lin

**Affiliations:** 1 Department of Chemical and Materials Engineering, National University of Kaohsiung, Kaohsiung City 811, Taiwan; qwe7711989@gmail.com; 2 Gerneral Manager Office, ThinTech Materials Technology Co., LTD., Kaohsiung City 811, Taiwan; ytpan2005@gmail.com

**Keywords:** heat-resistant steel, MX, precipitation, Z phase, Nb-doping

## Abstract

In this study, 22Cr25NiWCuCo(Nb) heat-resistant steel specimens with high Cr and Ni contents were adopted to investigate the effect of Nb content on thermal and precipitation behavior. Differential scanning calorimetry profiles revealed that the melting point of the 22Cr25NiWCuCo(Nb) steel specimens decreased slightly with the Nb content. After heat treatment at 1200 °C for 2 h, the precipitates dissolved in a Nb-free steel matrix. In addition, the Z phase (CrNb(C, N)) and MX (Nb(C, N), (Cr, Fe)(C, N), and NbC) could be observed in the Nb-containing steel specimens. The amount and volume fraction of the precipitates increased with the Nb content, and the precipitates were distributed heterogeneously along the grain boundary and inside the grain. Even when the heat treatment duration was extended to 6 h, the austenitic grain size and precipitates became coarser; the volume fraction of the precipitates also increased at 1200 °C. The Z phase, rather than the MX phase, became the dominant precipitates at this temperature.

## 1. Introduction

Austenitic heat-resistant steel exhibits a high creep resistance [1,2] and high-temperature corrosion [3,4]. Solution strengthening and precipitation strengthening constitute the primary techniques for strengthening the characteristics of such steel or alloys. These techniques entail the addition of alloy elements to impart M_23_C_6_, MX, Laves, and Z phases, as well as Cu-rich precipitates [5,6,7,8]. Sanicro 25 steel is considered an excellent engineering material that can be used in the high-pressure and high-temperature environments of high-efficiency power stations [9]. Sanicro 25 steel is composed of approximately 22% Cr, 25% Ni, and many alloys (W, Nb, Co, Mn, Si, Cu, and 0.2% excess Nb) that contribute to its hot corrosion resistance and stabilize and strengthen the austenite matrix [10,11,12]. Studies have reported that Sanicro 25 steel possesses high strength properties and high creep resistance because of the stable microstructure of the Z, Laves, and MX phases at high temperatures [13,14,15]. Zurek et al. presented a detailed report on the formation of precipitates of Sanicro 25 steel, including those in the form of a Z-(Nb,Cr)N phase, chromium nitride (Cr_2_N), a tungsten-rich phase, and a µ phase (F_e7_W_6_), at temperatures between 600 and 750 °C for up to 10,000 h [11]. Moreover, Li et al. studied the nucleation and growth of secondary NbCrN in 25Cr–20Ni–Nb–N steel during long-term aging at 700 °C, revealing that NbN can provide a favorable site for the nucleation of NbCrN and that NbN can dissolve to supply Nb and N for NbCrN growth [16,17,18,19]. However, the phase transformation mechanism with respect to the applied alloys, temperature, and time is unclear. Understanding the phase components from the solidus temperature to 1200 °C is beneficial for controlling the phase transformations during thermal processes.

Z-phase and MX precipitates are crucial in austenitic heat-resistant steel. Most studies have focused on precipitation behavior between 600 and 750 °C in long-term aging. Moreover, Nb is an essential alloy involved in precipitate nucleation. Controlling the heterogeneous primary precipitates (precipitation temperature >1200 °C) significantly influences the nucleation and growth of secondary precipitates during warm working. Therefore, in the present study, 22Cr25NiWCoCu steel specimens were doped with different contents of Nb and subjected to heat treatment at 1200 °C for 2–6 h; the resulting microstructures, precipitates, and phase compositions were investigated. Understanding the phase constitution at the high temperature of 1200 °C is crucial because high-temperature phases influence precipitation behavior below the solution temperature. The distribution, particle size, and volume fraction of the precipitates were also evaluated, and the thermal stabilization of the doped 22Cr25NiWCuCo(Nb) steel specimens was investigated through thermal analysis to determine the relationship between Nb doping and the precipitates.

## 2. Materials and Methods

Heat-resistant 22Cr25NiWCoCu steel was doped with different contents of Nb (0, 0.3, 0.6, and 0.9 wt.%); the steel was prepared through vacuum induction melting. The steel ingots were hot rolled at 1250 °C and finished at an end-rolling temperature of 950 °C, with a final reduction ratio of 80%. Subsequently, the hot-rolled plates were subjected to heat treatment at 1200 °C for 2 h, followed by water cooling to maintain the high-temperature microstructure. The compositions of the 22Cr25NiWCuCo(Nb) steel samples are presented in Table 1.

The 22Cr25NiWCuCo(Nb) steel specimens were subjected to thermal analysis performed using a differential scanning calorimeter (Netzsch DSC 404) to obtain their differential scanning calorimetry (DSC) profiles. The analysis was executed at a maximum temperature of 1450 °C and heating rate of 5 °C/min in an argon atmosphere with a flow rate of 100 mL/min. The microstructures and phases of the samples were examined using a field-emission electron probe microanalysis (FE-EPMA) system (JEOL JXA-8530F) equipped with instruments for energy-dispersive spectroscopy (EDX) and wavelength-dispersive spectrometry (WDS). The microstructure observation and WDS examination proceeded under an acceleration voltage of 15 keV. Field-emission transmission electron microscopy (FE-TEM)—executed using a JEM-2100F system (JEOL)—along with selected area electron diffraction (SAD) and EDX were adopted for fine precipitate identification. FE-TEM examination was carried out with an acceleration voltage of 200 keV. The TEM specimens were prepared using an advanced focused ion beam system (FEI Helios G3CX).

## 3. Results

**Figure 1:**
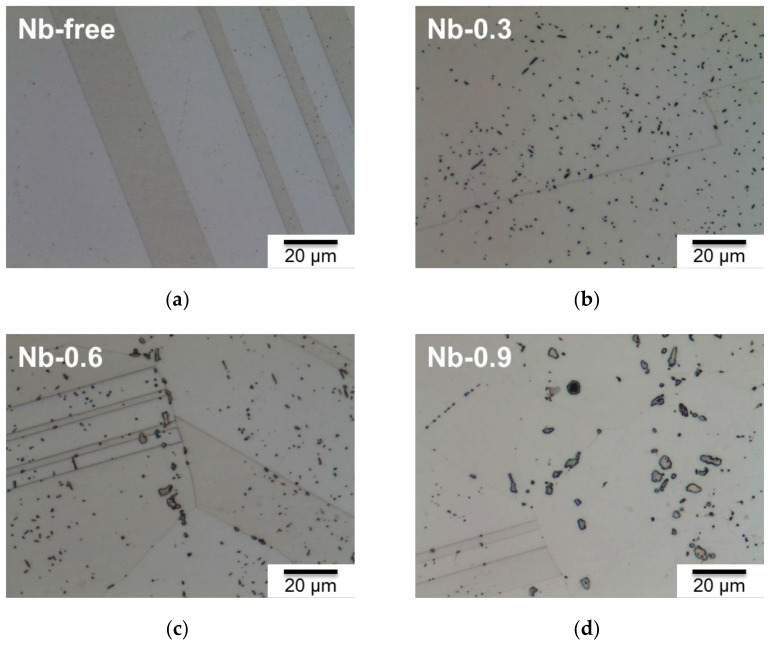
Optical microstructure and precipitate morphology of 22Cr25NiWCuCo(Nb) steel specimens after heat treatment at 1200 °C for 2 h: (**a**) Nb-free, (**b**) Nb-0.3, (**c**) Nb-0.6, and (**d**) Nb-0.9 steel specimens.

Figure 1 presents the microstructures and precipitate morphologies of the 22Cr25NiWCuCo(Nb) steel specimens after heat treatment at 1200 °C for 2 h. In the present study, precipitates in the Nb-free steel dissolved in the austenitic matrix after 2 h of heat treatment, as presented in Figure 1a. The precipitates could be observed after heat treatment, and the number of precipitates increased with the Nb content. Accordingly, in the present study, the microstructures had large amounts of heterogeneously distributed precipitates after 2 h of heat treatment at 1200 °C.

**Figure 2:**
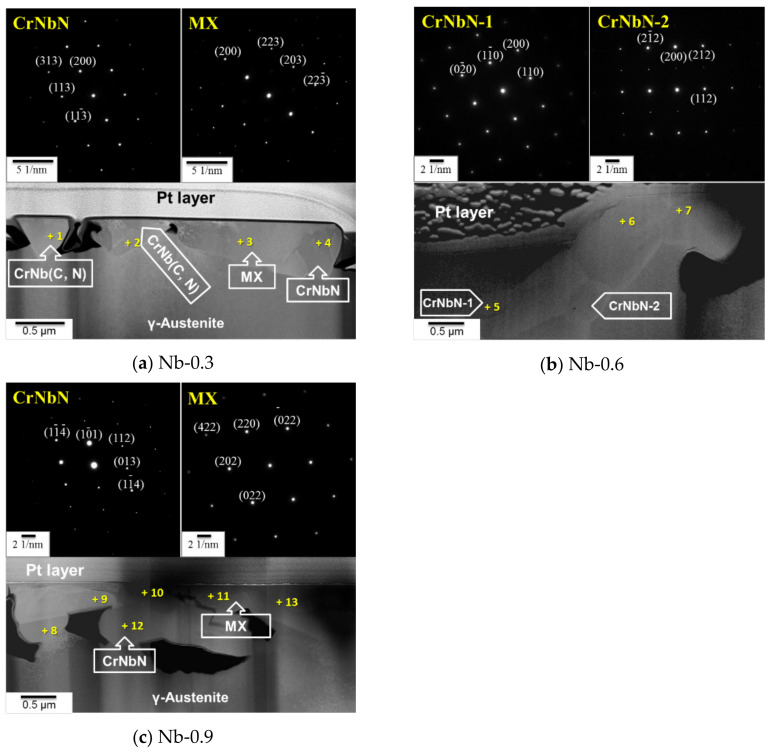
TEM–high-angle annular dark-field observation and analysis of 22Cr25NiWCuCo(Nb) steel specimens after solution treatment at 1200 °C for 2 h: (**a**) Nb-0.3, (**b**) Nb-0.6, and (**c**) Nb-0.9 steel specimens.

As presented in Figure 2, the precipitates aggregated in the matrix and along the grain boundary. High-resolution TEM (HRTEM)/SAD was used to identify the precipitates. The Nb-0.3 steel sample had a tetragonal CrNbN structure and an MX phase with an NaCl-type structure (Figure 2a). The HRTEM/EDS investigation of chemical composition indicated that the MX precipitate possessed a large amount of Nb reacting with interstitial C and N, as presented in Table 2. The Z phase with a (Cr, Nb)N composition also contained interstitial C parts, and the atomic ratio of C to N was approximately 1–1.2. The dominant precipitate in the Nb-0.6 steel specimen was CrNbN with a low C content (4.4–8.9 at.%), and the MX precipitate was not observed in TEM analysis. In addition, the Z phase and MX were the primary precipitates in the Nb-0.9 steel specimen, similar to the Nb-0.3 steel specimen. However, Nb and C appeared to be the predominant alloys in the MX in the Nb-0.9 steel specimen, similar to the NbC compound, as indicated in Figure 2c and Table 2. In Figure 2c, points 11 and 13 correspond to the MX phase; point 11 represents (Cr, Fe)(C, N), and point 13 represents NbC. However, whether the diffusion phenomenon between (Cr, Fe)(C, N) and NbC occurred with an increase in the isothermal time at a high temperature was unclear.

**Figure 3:**
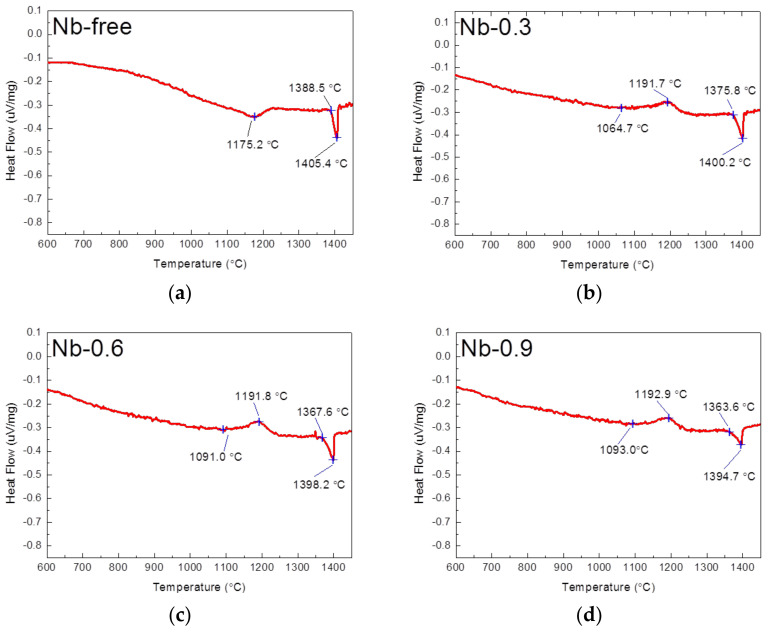
DSC profiles of the 22Cr25NiWCuCo(Nb) steel specimens obtained from thermal analysis: (**a**) Nb-free, (**b**) Nb-0.3, (**c**) Nb-0.6, and (**d**) Nb-0.9 steel specimens.

Figure 3 presents the DSC profiles of the 22Cr25NiWCuCo(Nb) steel specimens obtained from the thermal analysis. For the Nb-free steel specimen, the major endothermic peak started to form at 1388.5 °C—which is related to the melting point of steel, and it ended at the temperature of 1405.4 °C. When the Nb content was increased to 0.9 wt%, the temperature at which the major endothermic peak started to form decreased from 1388.5 to 1363.6 °C, and the temperature at which it peaked decreased from 1405.4 to 1394.7 °C. These findings indicate that the melting point of the 22Cr25NiWCuCo(Nb) steel specimens decreased slightly as the Nb content increased. Moreover, the Nb-free steel specimen had an endothermic peak at 1175.2 °C; by contrast, the 22Cr25NiWCuCo(Nb) steel specimens exhibited exothermic peaks at temperatures ranging between 1191.7 and 1192.9 °C. To determine the mechanisms underlying the formation of these peaks, further examination was performed.

**Figure 4:**
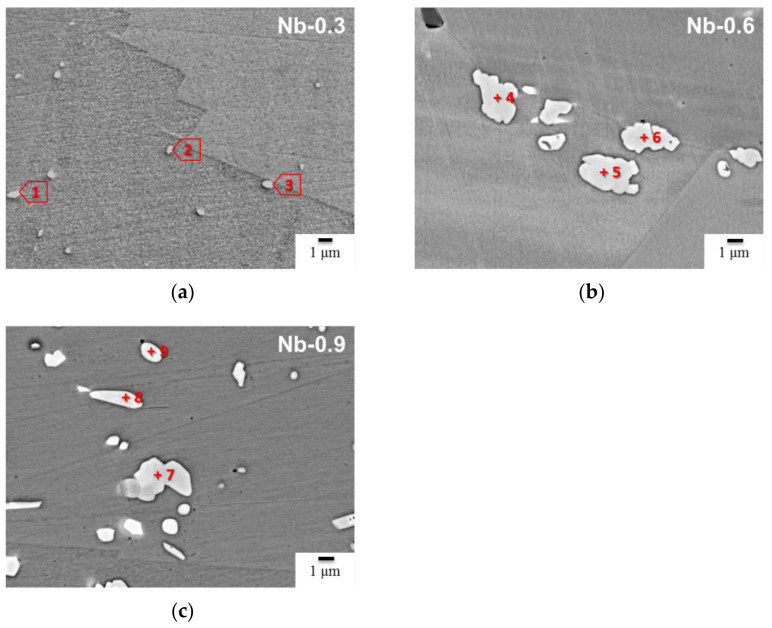
Microstructure and precipitate morphology of the 22Cr25NiWCuCo(Nb) steel specimens after heat treatment at 1200 °C for 6 h: (**a**) Nb-0.3, (**b**) Nb-0.6, and (**c**) Nb-0.9 steel specimens. (Marked points 1–9 is the chemical composition analysis which corresponding to Table 3.)

The duration of the heat treatment was extended to 6 h to observe the stable phases at 1200 °C and confirm phase transformation processes in subsequent aging treatments. As indicated in Figure 4, the precipitates maintained stabilized random distributions in the Nb-0.3, Nb-0.6, and Nb-0.9 steel specimens. After 6 h of heat treatment, the precipitates assembled into aggregation structures in the matrix and along the grain boundary; this behavior was the same as that observed in the 2 h heat treatment. Table 3 presents the FE-EPMA/WDX analysis results for the various phases in the 22Cr25NiWCuCo(Nb) steel specimens after heat treatment at 1200 °C for 6 h. As the precipitates in the Nb-0.3 steel specimen had a small particle size (<1 µm), distinguishing between the Z phase and MX was difficult. Although the probe size of the FE-EPMA/WDX system was approximately 0.5 μm, the matrix influenced the identification of the precipitate composition. Despite these difficulties, the analysis revealed that the C content was significantly lower than the nitrogen content in the Nb-0.3 steel specimen. Similar composition results were obtained for the Nb-0.6 and Nb-0.9 steel samples. Furthermore, the WDS analysis results revealed that the Z phase was the most dominant phase during treatment at 1200 °C. This finding suggests that the NbC carbide was completely dissolved into the matrix and that NbN gradually transformed into CrNbN during heat treatment at 1200 °C.

**Figure 5:**
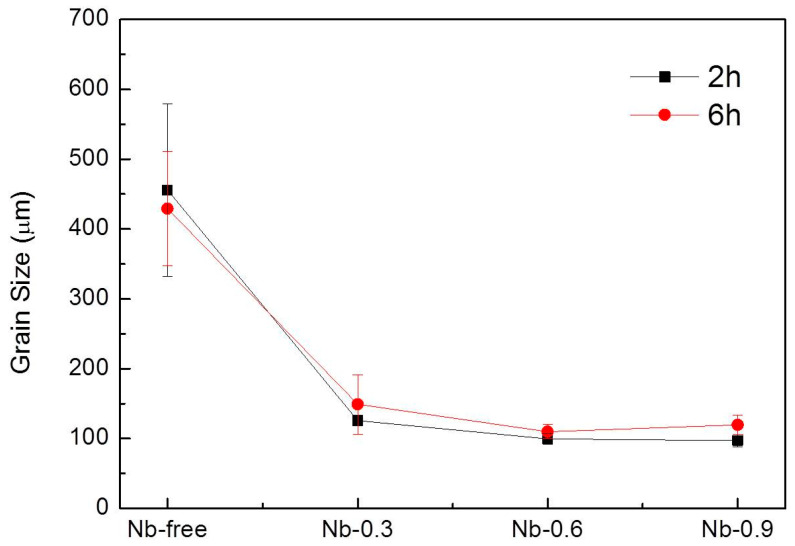
Average grain size as a function of Nb content in the 22Cr25NiWCuCo(Nb) steel specimens after heat treatment at 1200 °C for 2 and 6 h.

**Figure 6:**
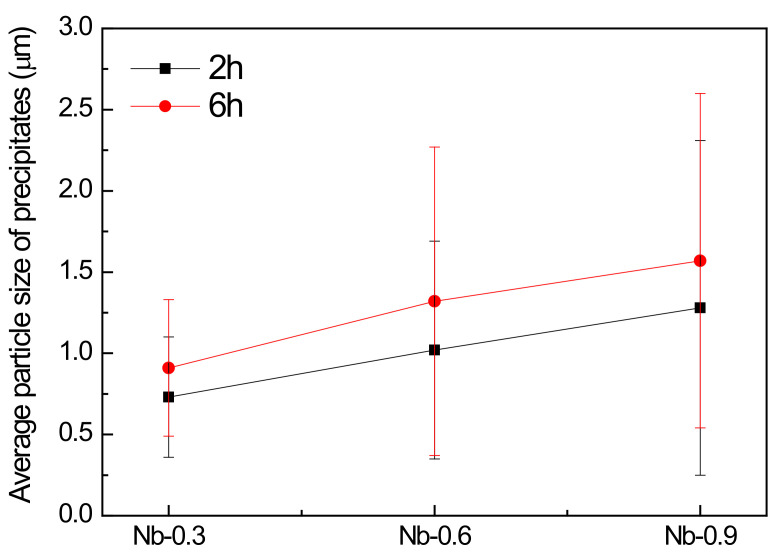
Average particle size of precipitates in the 22Cr25NiWCuCo(Nb) steel specimens with different Nb contents after heat treatment at 1200 °C for 2 and 6 h.

After 6 h of heat treatment at 1200 °C, the austenitic grains and precipitates became coarser. The average size of the austenitic grains decreased as the Nb content increased in both the 2 and 6 h heat treatment processes executed at 1200 °C. Compared with the measurements obtained after the 2 h heat treatment, the average grain size decreased by approximately 5.9% in the Nb-free steel sample and increased by approximately 10–23% in the Nb-doped steel samples after the 6 h heat treatment (Figure 5). As displayed in Figure 6, the average particle size of the precipitates increased from 0.7 ± 0.37 to 1.28 ± 1.03 µm as the Nb content increased. The 6 h heat treatment contributed to the increased average particle size, and the precipitate growth rate was 22–30%. As the precipitates assembled into aggregation structures, with each particle measuring hundreds of nanometers, quantifying the individual Z-phase and MX fractions was difficult. Therefore, the present precipitate quantification results are presented using the total Z and MX phases. An area of approximately 20 mm^2^ for each specimen was required for the quantitative calculation of the average austenitic grain size, particle size, and volume fraction of the precipitates.

**Figure 7:**
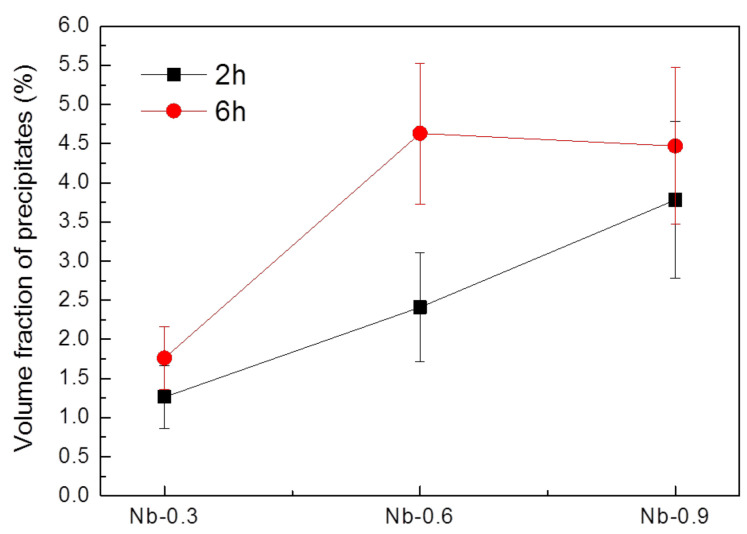
Volume fraction of precipitates in the 22Cr25NiWCuCo(Nb) steel specimens with different Nb contents after heat treatment at 1200 °C for 2 and 6 h.

**Figure 8:**
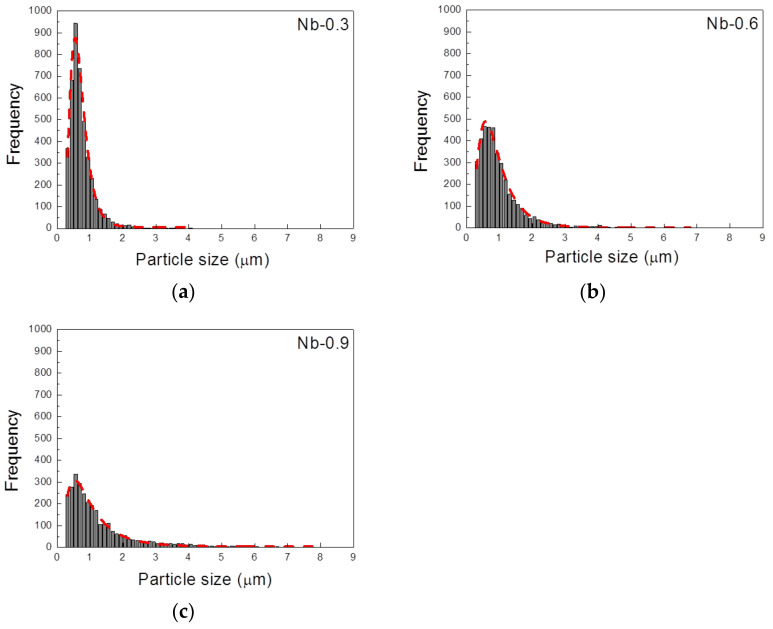
Histograms of precipitate size in the 22Cr25NiWCuCo(Nb) steel specimens with different Nb contents after heat treatment at 1200 °C for 2 h: (**a**) Nb-0.3, (**b**) Nb-0.6, and (**c**) Nb-0.9 steel specimens.

**Figure 9:**
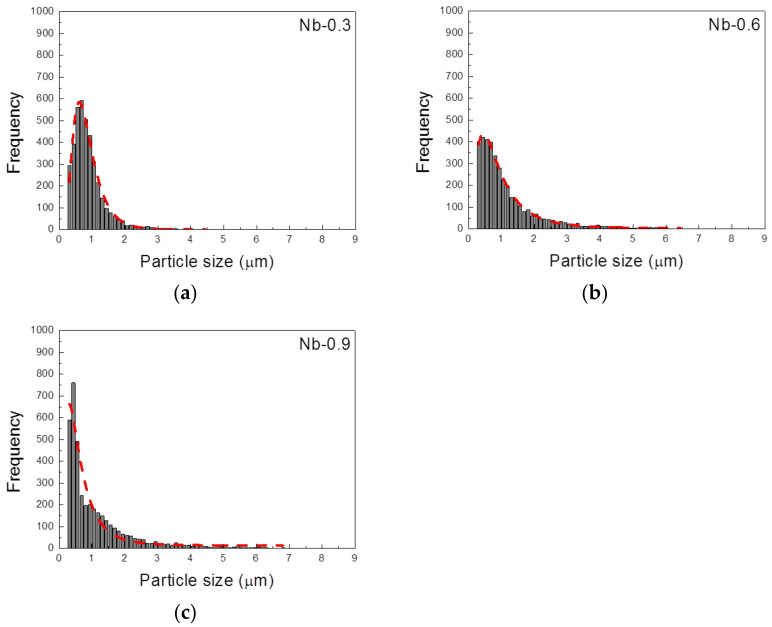
Histograms of precipitate size in 22Cr25NiWCuCo(Nb) steel specimens with different Nb contents after heat treatment at 1200 °C for 6 h: (**a**) Nb-0.3, (**b**) Nb-0.6, and (**c**) Nb-0.9 steel specimens.

Figure 7 illustrates the relationship between Nb content and the volume fraction of precipitates after 2 and 6 h of heat treatment. During the 2 h heat treatment, the volume fraction of the precipitates was positively correlated with the growth rate and Nb content. The volume fraction was 1.3% in the Nb-0.3 steel sample, 2.4% in the Nb-0.6 steel sample, and 3.8% in the Nb-0.9 steel specimen. The 6 h heat treatment contributed to the growth and coarsening of the MX precipitates, and the Z-phase precipitates increased in volume fraction. The volume fraction of the Nb-0.6 steel specimen was close to that of the Nb-0.9 steel specimen after the 6 h heat treatment. The histograms of precipitate size after heat treatment at 1200 °C for 2 h (Figure 8) indicate that the Nb-0.3 steel specimen had numerous fine precipitates concentrated in the size range of 0.4–0.8 µm. This result differed from the uniform distributions of precipitates in the Nb-0.6 and Nb-0.9 steel specimens, signifying that high Nb content can promote the nucleation and growth of the MX and Z phases. The particle size distribution curve of the precipitates in the Nb-0.3 steel specimen after 6 h of heat treatment indicates that numerous particles with a size of 0.5 µm coarsened, resulting in a broad distribution curve (Figure 9a). This phenomenon of coarsening precipitates was also observed in the Nb-0.6 steel specimen (Figure 9b). However, the number of particles measuring 0.3–0.5 µm in the Nb-0.9 steel specimen abruptly increased after 6 h of heat treatment, meaning that numerous fine precipitates formed in this specimen (Figure 9c).

## 4. Discussion

The precipitates morphology of the 22Cr25NiWCuCo(Nb) steel specimens also appeared to become coarser as the Nb content increased, as shown in Figure 1. In the steel specimens containing Nb, a sufficient amount of free Nb had a high affinity to react with C or N that was available in the solid solution, resulting in precipitates such as Z-phase, MX carbonitride, M_23_C_6_, and Cu-rich precipitates [20,21,22]. Many studies have demonstrated that the grain boundary has higher interfacial energy than the grain does and provides a faster diffusion path for alloying [8,23,24]. Therefore, the nucleation and growth of different types of precipitates have been observed along the grain boundary and even inside the grains. From the chemical composition of precipitates that could be observed, the Z phase with a (Cr, Nb)N composition also contained interstitial C parts in Nb-0.3 and Nb-0.6 steels, where Raghavan et al. has also discussed the dissolution of carbon in CrNbN to form CrNb(C, N) [25]. However, the MX became one of the predominant phases in the Nb-0.9 steel specimen because Nb-0.9 steel contained the higher Nb content. Sourmail argued that both the Z phase and NbC can be present because of the sufficient amount of Nb [19].

The DSC profiles of the 22Cr25NiWCuCo(Nb) steel specimens obtained from the thermal analysis showed that the Nb-free steel specimen had an endothermic peak at 1175.2 °C, and the 22Cr25NiWCuCo(Nb) steel specimens exhibited exothermic peaks at temperatures ranging between 1191.7 and 1192.9 °C. Specifically, the fine precipitates formed in the Nb-free steel specimen were examined using field-emission scanning electron microscopy and EDS, which revealed that oxides, carbides, and other impurities were the major compounds [19]. The precipitates and impurities dissolved in the matrix were presumed to have caused an exothermic reaction, which thus resulted in the endothermic peak observed for this specimen. Additionally, the endothermic reaction specimens underwent endothermic reactions at 1191.7–1192.9 °C, resulting in the formation of the MX and CrNb(C)N phases; these could have engendered the exothermic peaks observed for these specimens. According to the literature [19,25,26], the Z phase in austenitic stainless steel is stable at temperatures below 1300 °C. The Z phase is also a predominant precipitate at a wide range of temperatures from 600 to 1200 °C [27]. As reported by Sourmail [19] and Heczko et al. [28], a high-temperature solid solution is an essential prerequisite for phase transformation among the Z phase, Nb(C, N), NbN, and NbC. High-temperature treatment may thus have contributed to the nucleation and growth of the MX and Z-phase precipitates along the grain boundary and inner grain [29], as illustrated in Figure 1 and Figure 2.

From the results of the average variation in austenitic grain size and average particle size of precipitates after 2 and 6 h of heat treatment at 1200 °C (as shown in Figure 5 and Figure 6), the 6 h heat treatment contributed to the increased average particle size and the precipitate growth rate. It indicates that precipitates hinder grain boundary movement, preventing grain growth and maintaining thermal stability [30]. These findings also show that the endothermic reaction between 1191.7 and 1192.9 °C in the 22Cr25NiWCuCo(Nb) steel specimens was beneficial for the coarsening of the MX and Z-phase precipitates. Despite the volume fraction and average particle size of the precipitates, it was positively correlated with the growth rate and Nb content during the 2 h heat treatment at 1200 °C. It was noticed that the volume fraction of the Nb-0.6 steel specimen was close to that of the Nb-0.9 steel specimen after the 6 h heat treatment. Moreover, the numerous fine precipitates formed with a size of approximately 0.4 µm in the Nb-0.9 steel specimen abruptly increased after 6 h of heat treatment, which caused the volume fraction of the precipitates to remain constant after 6 h. Ghosh [31] and Zhou et al. [32] have demonstrated that increasing the isothermal temperature can increase the size and coarsening rate for carbonitrides. A high isothermal temperature increases the diffusion rate of atoms to reduce the number of carbonitride particles, increasing the distance among the growing particles. Even if the solute and matrix concentrations are gradually reduced, the driving force for the diffusion of interfacial solute atoms is reduced [33]. As the diffusion distance is directly proportional to time and the size variation of carbonitrides exhibits a parabolic relationship with aging time, the coarsening rate of MX and Z-phase precipitates is time-independent [32]. Therefore, the coarsening rate and volume fraction of the precipitates in the Nb-0.9 steel specimen after 6 h of heat treatment were reduced compared with those of the precipitates in the Nb-0.6 steel specimen.

## 5. Conclusions

In this study, hot-rolled plates of 22Cr25NiWCuCo(Nb) heat-resistant steel were subjected to heat treatment at 1200 °C for 2 h to investigate the effect of Nb content on the precipitates formed in and thermal behavior of the plates and to understand the dominant phase constitution during treatment. The results reveal that the Z phase (CrNb(C, N)) and MX (including Nb(C, N), (Cr, Fe)(C, N), and NbC) were the dominant precipitates in the 22Cr25NiWCuCo(Nb) steel specimens. A high Nb content (0.9 wt%) in 22Cr25NiWCuCo(Nb) steel was determined to be beneficial for NbC formation as the Nb content reached 0.9 wt%. After the heat treatment, the Nb-free steel specimen exhibited a nonprecipitated microstructure that was dependent on the Nb content. This thus signifies that a high Nb content is beneficial for the precipitation of NbC. Moreover, increasing the content of Nb is advantageous for suppressing grain growth at high temperatures because it promotes precipitate formation. The specimens’ DSC profiles revealed that the melting point of the 22Cr25NiWCuCo(Nb) steel samples decreased slightly as the Nb content increased. Extending the heat treatment duration to 6 h also promoted the coarsening and increased the volume fraction of the precipitates at 1200 °C. The Z phase replaced the MX to become the dominant precipitates at 1200 °C.

In steel manufacturing, homogenous treatment (>1200 °C) is an important process to obtain a uniform microstructure. The heterogeneous primary precipitates could influence the nucleation and growth of secondary precipitates during warm working. Moreover, understanding the Nb alloying addition and establishment of the Z-phase, MX, carbide precipitation mechanism at high temperature is beneficial to mechanical property relations. Therefore, the high-temperature deformation behavior of 2Cr25NiWCuCo(Nb) steel is future research.

## Figures and Tables

**Table 1:** Compositions of 22Cr25NiWCuCo(Nb) steel specimens (wt.%).

	Cr	Ni	W	Cu	Co	N	C	Si	Mn	P	S	Nb	Fe
Nb-free	22.20	24.40	3.39	2.90	1.47	0.23	0.07	0.19	0.49	0.009	0.005	-	Bal.
Nb-0.3	22.10	24.30	3.40	2.90	1.49	0.23	0.07	0.19	0.49	0.010	0.005	0.29	Bal.
Nb-0.6	21.90	24.20	3.36	2.90	1.45	0.23	0.07	0.20	0.49	0.010	0.005	0.58	Bal.
Nb-0.9	21.80	24.10	3.52	2.90	1.54	0.23	0.07	0.20	0.49	0.010	0.005	0.86	Bal.

**Table 2:** High-resolution TEM (HRTEM)/EDX analysis of different phases in 22Cr25NiWCuCo(Nb) steel specimens after heat treatment at 1200 °C for 2 h (for the marked points in Figure 2).

	Cr at.%	Fe at.%	Nb at.%	W at.%	C at.%	N at.%	Si at.%	Phase
1 (Nb-0.3)	28.5 ± 0.9	5.5 ± 0.3	31.9 ± 1.6	2.5 ± 0.8	16.0 ± 0.8	15.6 ± 0.5	-	CrNb(C, N)
2 (Nb-0.3)	26.2 ± 0.9	4.9 ± 0.3	29.1 ± 1.6	1.9 ± 0.9	20.8 ± 0.9	17.1 ± 0.6	-	CrNb(C, N)
3 (Nb-0.3)	5.6 ± 0.3	3.4 ± 0.2	47.8 ± 1.4	-	27.0 ± 1.0	16.2 ± 0.5	-	MX
4 (Nb-0.3)	30.5 ± 1.0	4.0 ± 0.3	33.6 ± 1.6	1.3 ± 0.9	-	30.6 ± 0.5	-	CrNbN
5 (Nb-0.6)	29.8 ± 1.0	6.7 ± 0.3	30.2 ± 1.5	1.6 ± 0.8	8.9 ± 0.8	22.8 ± 0.6	-	CrNb(C, N)
6 (Nb-0.6)	29.3 ± 0.9	3.9 ± 0.2	30.7 ± 1.4	1.7 ± 0.6	4.4 ± 0.4	30.0 ± 0.5	-	CrNb(C, N)
7 (Nb-0.6)	27.1 ± 0.8	5.1 ± 0.3	34.1 ± 1.6	1.8 ± 0.9	4.9 ± 0.6	27.0 ± 0.5	-	CrNb(C, N)
8 (Nb-0.9)	30.1 ± 0.9	5.3 ± 0.3	30.5 ± 1.6	1.3 ± 0.9	15.2 ± 0.8	17.6 ± 0.6	-	CrNb(C, N)
9 (Nb-0.9)	24.9 ± 0.8	4.4 ± 0.3	30.3 ± 1.5	1.4 ± 0.8	30.1 ± 1.0	8.9 ± 0.5		CrNb(C, N)
10 (Nb-0.9)	28.4 ± 0.9	4.5 ± 0.3	31.1 ± 1.6	1.4 ± 0.9	25.6 ± 0.9	7.3 ± 0.5	1.7 ± 0.6	CrNb(C, N)
11 (Nb-0.9)	36.1 ± 1.2	17.5 ± 0.6	3.0 ± 0.6	1.3 ± 0.5	22.7 ± 1.0	19.4 ± 0.5		MX
12 (Nb-0.9)	33.3 ± 1.1	5.3 ± 0.2	29.4 ± 1.4	1.9 ± 1.0	12.6 ± 0.7	16.3 ± 0.6	1.2 ± 0.6	CrNb(C, N)
13 (Nb-0.9)	3.0 ± 0.2	1.6 ± 0.3	46.7 ± 1.8	-	41.9 ± 1.2	4.0 ± 0.3	2.8 ± 0.8	MX

**Table 3:** EPMA/WDX analysis of different phases in the 22Cr25NiWCuCo(Nb) steel specimens after heat treatment at 1200 °C for 6 h (for the marked points in Figure 4).

	Cr at.%	Fe at.%	Nb at.%	W at.%	C at.%	N at.%	Cu at.%	Ni at.%	Co at.%	Mn at.%
1 (Nb-0.3)	29.3	27.6	14.9	1.3	3.3	7.5	1.2	13.7	0.8	0.4
2 (Nb-0.3)	25.8	36.6	5.4	1.2	1.6	6.5	1.8	19.4	1.2	0.5
3 (Nb-0.3)	23.8	34.9	5.6	1.0	2.1	11.1	1.6	18.2	1.2	0.5
4 (Nb-0.6)	29.1	4.5	29.5	1.3	4.2	30.3	0.1	0.8	0.1	0.1
5 (Nb-0.6)	28.7	4.5	30.2	1.4	4.0	30.2	-	0.8	0.1	0.1
6 (Nb-0.6)	28.8	4.3	32.0	1.5	4.5	28.1	-	0.6	0.1	0.1
7 (Nb-0.9)	27.3	4.5	28.6	1.3	2.9	34.2	-	0.9	0.2	0.1
8 (Nb-0.9)	28.6	5.2	29.1	1.4	2.9	31.4	-	1.1	0.1	0.2
9 (Nb-0.9)	27.0	12.4	22.9	1.3	2.9	27.8	0.4	4.9	0.3	0.1

## Data Availability

Data sharing is not applicable to this article.
